# A cost-benefit analysis of a proposed overseas refugee latent tuberculosis infection screening and treatment program

**DOI:** 10.1186/s12889-015-2530-7

**Published:** 2015-12-01

**Authors:** La’Marcus T. Wingate, Margaret S. Coleman, Christopher de la Motte Hurst, Marie Semple, Weigong Zhou, Martin S. Cetron, John A. Painter

**Affiliations:** Division of Global Migration and Quarantine, Centers for Disease Control and Prevention, Atlanta, GA USA

**Keywords:** Latent tuberculosis, Screening, Refugees, Cost-benefit analysis, Rifapentine

## Abstract

**Background:**

This study explored the effect of screening and treatment of refugees for latent tuberculosis infection (LTBI) before entrance to the United States as a strategy for reducing active tuberculosis (TB). The purpose of this study was to estimate the costs and benefits of LTBI screening and treatment in United States bound refugees prior to arrival.

**Methods:**

Costs were included for foreign and domestic LTBI screening and treatment and the domestic treatment of active TB. A decision tree with multiple Markov nodes was developed to determine the total costs and number of active TB cases that occurred in refugee populations that tested 55, 35, and 20 % tuberculin skin test positive under two models: no overseas LTBI screening and overseas LTBI screening and treatment. For this analysis, refugees that tested 55, 35, and 20 % tuberculin skin test positive were divided into high, moderate, and low LTBI prevalence categories to denote their prevalence of LTBI relative to other refugee populations.

**Results:**

For a hypothetical 1-year cohort of 100,000 refugees arriving in the United States from regions with high, moderate, and low LTBI prevalence, implementation of overseas screening would be expected to prevent 440, 220, and 57 active TB cases in the United States during the first 20 years after arrival. The cost savings associated with treatment of these averted cases would offset the cost of LTBI screening and treatment for refugees from countries with high (net cost-saving: $4.9 million) and moderate (net cost-saving: $1.6 million) LTBI prevalence. For low LTBI prevalence populations, LTBI screening and treatment exceed expected future TB treatment cost savings (net cost of $780,000).

**Conclusions:**

Implementing LTBI screening and treatment for United States bound refugees from countries with high or moderate LTBI prevalence would potentially save millions of dollars and contribute to United States TB elimination goals. These estimates are conservative since secondary transmission from tuberculosis cases in the United States was not considered in the model.

**Electronic supplementary material:**

The online version of this article (doi:10.1186/s12889-015-2530-7) contains supplementary material, which is available to authorized users.

## Background

In the United States, most active tuberculosis (TB) cases occur in foreign-born residents [1], and over 80 % of these cases occur in persons thought to have acquired latent tuberculosis infection (LTBI) overseas [2]. This epidemiologic pattern has strongly influenced CDC’s determination that identifying and treating LTBI is an important public health intervention to reduce the prevalence of active pulmonary TB cases [3].

Persons seeking entry into the United States for permanent residence (immigrants and refugees) receive a mandatory overseas medical exam and are treated for communicable diseases of public health significance such as active, infectious TB prior to departure [4]. Since LTBI is non-infectious, treatment for LTBI is not currently required. Medical exams are administered by panel physicians. Panel physicians work in the immigrants’ and refugees’ country of origin in most instances and are extended permission from the consular sections of United States embassies to administer medical exams that meet CDC standards (Technical Instructions) [5].

As a method for finding active TB in children under age 15 who live in countries with TB prevalence greater than 20 per 100,000, LTBI testing is performed. Those with negative tests do not undergo further TB screening. Immigrants and refugees age 15 and older (adults) are not required to be tested for LTBI in their medical exams, but they do receive mandatory chest radiographs as a means of testing for active TB [5]. Refugees and immigrants with an abnormal chest radiograph, but negative results from sputum-smear microscopy for acid-fast bacilli and culture for *Mycobacterium tuberculosis*, are given a Class B1 TB classification that helps prioritize them for further TB-related screening once they reach the United States [6].

The result of this age-specific screening is that a majority of the approximately 500,000 immigrants and refugees resettled annually are examined for active TB and if diagnosed are treated prior to arriving in the United States; however, they are not tested for LTBI overseas. Therefore, this population of permanent entrants represents a potential reservoir of LTBI that can lead to TB cases [2, 7, 8].

Immigrants pay for their own medical examinations and treatments overseas, whereas the United States government pays the cost of refugees’ exams. The United States government also pays for healthcare for at least the first 8 months after a refugee’s arrival, and in many states refugees are eligible for Medicaid for some time past the first 8 months [9]. To address the long-term impact of reactivated TB (progression from LTBI to active TB), CDC has been considering adding voluntary LTBI screening and treatment for adult refugees and immigrants to the current screening for active TB that is already a part of the TB Technical Instructions issued to panel physicians. In this evaluation, we focus on refugees, in part because they typically have a longer interval between examination and departure for resettlement in the United States than do immigrants. The overseas refugee medical screenings take place 2 to 6 months before departure (Dr. Tarissa Mitchell, personal communication), so overseas LTBI treatment of less than 6 months’ duration could be completed without delaying resettlement. Such a proposed voluntary overseas LTBI program is made feasible by a new drug regimen. Until recently, standard LTBI treatment consisted of a daily dose of isoniazid for 9 months [10]. In 2011, CDC recommended use of a 12-dose weekly isoniazid-rifapentine regimen (3HP) to treat LTBI [11, 12]. The 12-dose regimen may be advantageous because it is administered weekly (with directly observed therapy [DOT]) rather than daily, and treatment is 3 months rather than 9 months in duration.

Compliance with voluntary LTBI treatment may be higher for refugees living overseas than for those who have resettled in the United States. While overseas, refugees are able to direct much of their attention towards receiving clearance to enter the United States. After arrival, they have competing priorities (e.g., to find a home, a job, enroll children into a school, learn a new language, and generally acculturate to a new community) [13]. There is also some evidence of the success of other voluntary medical protocols; for example, an overseas program of presumptive intestinal parasite treatment in refugees has had a high participation rate [14].

After resettlement, all refugees are encouraged to receive a general follow-up medical exam at their local public health department (PHD) which may include tests for LTBI and an opportunity for treatment [15]. In addition, PHDs are notified of refugees in their jurisdiction who had an abnormal chest radiograph (1–15 % of the population, depending on country of birth) during the overseas exam so that these individuals may be prioritized for TB-related testing [6, 16]. However, only about 75 % of recently arrived refugees receive this follow-up screening [7], and there is limited data to suggest that screening practices vary across the country, so that LTBI screening may be performed more frequently in some jurisdictions than others [17–19]. Once they have arrived in the United States, nearly a quarter of those diagnosed with LTBI decline therapy, and about 70 % of refugees who start 9 months of isoniazid treatment complete the therapy [20–23]. Therefore, a voluntary overseas LTBI testing and treatment program could be expected to have a higher rate of enrollment and completion than the current alternative.

We propose to analyze a voluntary overseas LTBI testing and treatment program by estimating the costs and benefits of two models : a new protocol of screening and treating all adult refugees for LTBI while still overseas, and the current protocol of domestic LTBI screening and treatment.

IRB review and approval was not required. This study was determined by CDC and Emory University to be research not involving human subjects.

## Methods

### Model data

This analysis compared the costs and benefits of the current domestic and proposed overseas programs to screen for and treat LTBI. In both programs, we assume that LTBI screening is performed using the tuberculin skin test (TST), because it is the least expensive and most widely available test. From the literature, we obtained ranges of the proportion of both immigrants and refugees with positive TST rates settling in the United States [24–27]. We assumed that these data would provide possible ranges for the rates of positive TSTs in U.S.-bound refugees.

In 2012 the majority of refugees that resettled in the United States were screened in nine countries: Ethiopia, Kenya, Uganda, Iraq, Jordan, Syria, Malaysia, Nepal, and Thailand [28]. For our analysis we categorized refugees into three hypothetical cohorts of 100,000 with high (55 %), moderate (35 %), and low (20 %) rates of positive TST results [24–27]. The respective categories reflect how often the refugees test TST positive in relation to other refugee populations. These TST results and the sensitivity and specificity of the TST were used to estimate LTBI prevalence. The estimate of LTBI prevalence was then used to estimate the number of active TB cases which might occur after domestic resettlement. The estimated total program costs included treatment of active TB cases and resources used to screen for and treat LTBI, including TSTs, DOT labor, and medications.

We used data from the International Organization for Migration to determine the prevalence of active TB in refugee populations from the nine countries [28]. Countries were divided into high, moderate, and low prevalence of active TB based upon the following thresholds: high (at least 600 cases per 100,000), medium (between 100 to 600 cases per 100,000) and low (less than 100 cases per 100,000). We assumed that the number of adults treated for active TB during overseas screening would be equal to the averages of active TB found within each of these three regions.

### Model overview

A decision tree model with Markov nodes was used to estimate the comparative costs and benefits of the current domestic LTBI protocol with the proposed overseas LTBI screening and treatment protocol. The model was developed using TreeAge Pro Software (Williamstown, MA, U.S.). For each protocol, the model estimated the number of refugees from each hypothetical cohort that would develop active TB during a 20-year period after resettlement. Our primary measure was the net benefit or cost from implementing overseas screening programs which was calculated using the following formula:

$$ Total\  program\  cost\  with\  no\  overseas\  screening\  and\  treatment\ \hbox{--}\ Total\  program\  cost\  with\  overseas\  screening\  or\  treatment. $$

The refugees entered into the decision tree were assumed to have one of three TB conditions: active TB, LTBI, or uninfected. First we modeled the current protocol where refugees are screened for LTBI after arrival in the United States, and if positive, are offered treatment at the local PHD [15]. Then, we modeled the proposed overseas protocol that adds a TST to the mandatory chest radiograph already administered to adults during pre-departure medical exams [5]. If the TST was positive, and if there were no indications of active TB in the chest radiograph, the adult refugee was assumed to be diagnosed with LTBI and was offered treatment to be completed prior to resettlement.

This analysis was conducted from both government and health systems perspectives. All costs were presented in 2012 US dollars. Further, a 3 % annual discount rate was used for all costs and health outcomes in accordance with recommendations for economic evaluations of healthcare programs [29]. Discounting was used to calculate the present value of costs and benefits which occurred after the first year of the model. In our base case analysis, we used a 20-year analytic horizon. Background all-cause mortality rates, grouped in 5-year increments (e.g., 30–34, 35–39), were incorporated using CDC data from 2005–2010 for the general US population [30].

For the model, we needed estimates of LTBI “prevalence.” We estimated the prevalence from the percentage of the population testing positive with the TST:

- (% TST test positive + specificity – 1)/(TST sensitivity + specificity −1) [31].
  - TST sensitivity was 89 %, an estimation that has been used in CDC guidelines [32].

- We assumed an average specificity of 85 % in our model because [33]:
  - Inoculation with Bacillus Calmette-Guerin (BCG) can affect the specificity of TST [34];
  - Refugees who have received BCG vaccine once in infancy test at 92 % specificity with the TST, while those who have not received the vaccine test at 98 % specificity [34];
  - Those receiving BCG after infancy test at 60 % specificity with the TST [34].

Since we had no way of determining how many adult refugees had received BCG vaccination during or after infancy, we assumed that one-third fit into each category, with an average specificity of approximately 85 %.

### Current protocol- domestic testing and treatment of refugees with LTBI

In the current protocol, adult refugees are not screened for LTBI overseas, but rather during post-domestic resettlement. However, refugees are tested for active disease during overseas screening, and in a manner similar to a prior economic evaluation, we assume that all individuals with active TB overseas will have an abnormal chest radiograph [33]. All individuals diagnosed with active TB overseas are treated prior to U.S. arrival [5]. We also assume that among our cohort with TB infection, 11 % will have inactive disease and an abnormal chest radiograph [33]. Due to imperfect specificity of the chest radiograph, a small portion of those with no infection (5 %) will also have abnormal chest radiographs [35]. After resettlement, a small portion of those with abnormal chest radiographs (1.5 %) will be diagnosed with active TB within their first year of arrival, and we also included this in our model [36].

We estimated that 76 % of all refugees present for domestic medical exams at PHDs where the cost of a TST will be incurred for those undergoing LTBI screening [7, 37]. The figure of 76 % is based upon data from a study that evaluated how often PHDs submit forms to the CDC indicating that they had performed any type of follow-up evaluation among Class B1 refugees. The lower bound estimate of 62.3 % was based upon the number of completed forms received by CDC indicating that the follow-up evaluation had been completed, and the upper bound estimate of 90.4 % was based upon the assumption that the follow-up had been completed although the documentation was not sent into the CDC.

The CDC maintains records of the tests performed during follow-up examinations and stores the results of these tests in the Electronic Disease Notification System (EDN) for refugees with a Class B1 designation [6]. From a national perspective, unpublished EDN data indicated that about 75 % of Class B1 refugees who presented for their domestic medical exam received LTBI testing (Dr. John Painter, personal communication), however the rate of testing is likely to vary across jurisdictions [17–19]. It is likely that individuals who had a Class B1 TB classification overseas received LTBI testing more often than those with no TB classification, as PHDs seem to place greater priority on testing those at higher risk of developing active disease. We used varying levels of domestic LTBI testing in our model due to uncertainty in the extent of domestic LTBI testing and in order to present more nationally representative results. In situations where LTBI examinations occur frequently, we assume 100 % LTBI testing for Class B1 refugees and 75 % LTBI testing for refugees with no TB classification. To model situations where LTBI testing occurs at a moderate rate, we assumed 75 % LTBI testing for Class B1 refugees and 50 % LTBI testing for refugees with no TB classification. In scenarios where LTBI testing occurs more infrequently, we assumed that 50 % of Class B1 refugees receive LTBI testing and 25 % of refugees with no TB classification receive LTBI testing.

Based upon previous research, we assumed that 77 % of those who received post-arrival medical screening and were diagnosed with LTBI accepted treatment in our model [38]. Domestic LTBI treatment was modeled with the 3HP regimen, which had documented completion rates of 82 % [11]. We estimated that the medication would be 93 % effective [39] in preventing future progression to active TB. For the 18 % of individuals who did not complete treatment, we assumed that they completed 2 weeks of the regimen, with a corresponding effectiveness of 0 % [39].

Refugees who did not present for follow-up or declined domestic treatment for LTBI, and those who did not complete treatment were assumed to have a 0.1 % annual probability of developing active TB disease [33, 40].

For the purposes of this model, no refugee diagnosed and treated for active TB overseas received a TST at domestic follow-up. This is because domestic PHDs interpret documentation of prior TB treatment as an indication that TSTs for LTBI would not be required. Thus, refugees treated for active TB overseas incurred no additional domestic costs related to LTBI screening or treatment.

### Proposed protocol—overseas testing and treatment of refugees with LTBI

The proposed protocol added a TST to the current medical exam administered to all adult refugees before they entered the United States. Since all adults currently receive a chest radiograph, the addition of LTBI testing is not likely to change the sensitivity of overseas screening for active TB. Individuals diagnosed with active TB disease are treated the same in the current and proposed protocols. Even though relapse could occur after individuals are treated for active disease overseas [41], the magnitude of this effect would not differ between the proposed and current protocols. Thus, these cases are not included in the estimates of TB cases occurring in the United States. Refugees diagnosed with LTBI through a positive TST result were offered the 3HP regimen prior to leaving for the United States.

We assumed that 95 % of refugees diagnosed with LTBI overseas would accept 3HP treatment, and the only reason refugees would fail to complete treatment was because of side effects (5 %) [11]. We based this on the fact that voluntary programs with presumptive treatment of intestinal parasites for refugees while overseas have high acceptance and completions rates [14]. We assumed that refugees who ceased treatment did so after completing 2 weeks of the regimen with a corresponding effectiveness of 0 % [39].

As a follow up to the overseas protocol, we assumed that 76 % [7] of refugees would present for domestic follow-up in PHDs. The only refugees who would need testing for LTBI during domestic follow-up would be those with a negative TST overseas, because everyone else with a positive TST would have either been treated overseas, or would have already been ruled out for 3HP due to medical contraindications. We did not adjust for a booster effect in follow-up TST testing as the second testing takes places months after the overseas testing. To the extent that this occurs, it would lead to more false positive being treated, but would not affect the number of cases prevented. Once again, in our base case, refugees who were Class B1 overseas would have a 75 % chance of receiving LTBI testing at follow-up, and those with no TB classification would have a 50 % chance of receiving LTBI testing at follow-up.

If refugees tested TST-positive in the United States, they would be offered domestic 3HP treatment. Of those refugees testing positive, approximately 77 % [38] would accept treatment, and 82 % of those starting treatment would complete it [11]. For the 18 % of individuals who did not complete treatment, we assumed that they completed 2 weeks of the regimen, with a corresponding effectiveness of 0 % [39].

Whether overseas or domestic, completion of treatment with 3HP was assumed to have an efficacy of 93 % [39] in preventing progression to active TB disease as estimated in a previous economic evaluation. Refugees who did not present or declined domestic treatment for LTBI and those who did not complete treatment were assumed to have a 0.1 % annual probability of developing active TB [33, 40].

### Cost overview

All costs were reported in 2012 United States dollars, and were calculated from the health system and government perspectives. Overseas and domestic costs included TST kit prices, labor to administer 3HP DOT, and isoniazid and rifapentine drug prices. Domestic costs also included treatment for active TB cases. Drug costs were the same in both domestic and international settings.

### Costs of TST

For overseas estimates of TST prices, we first obtained TST prices from a panel physician in Kenya as a base observation. We used purchasing power parity (PPP) to estimate what the price of the TST would be in other countries included in the study [42]. Dividing the PPP by the market exchange rate yields a national price level which can be used to compare the price of a basket of goods in one country with a reference country. For example, because Kenya had a national price level of 0.44, $0.44 spent in the Kenya purchased the same amount of goods as $1.00 spent in the United States. We obtained national price levels from the World Bank [42], and used those to estimate the costs of TSTs in the other countries in our study where refugees came from, although data was not available from Syria. We averaged the price from the remaining eight studies to obtain an estimate of $4.50 for the cost of providing a TST to these refugees overseas. For domestic estimates of TST prices, we referred to the Physician’s Fee and Coding Guide and used the lowest listed price to represent the costs of a TST [43].

### Wages and DOT labor costs

The country-specific cost for labor to administer 3HP was determined using the most recently available (2011–2013) United Nations (UN) pay scales at the time the study was conducted. Each worker was assumed to be compensated according to the UN General Service Category step 3 level 3 pay grade [44]. Five of the nine countries had wage data corresponding to 2012, and we adjusted wages for the other countries to 2012 using GDP deflator data obtained from the International Monetary Fund [45]. We converted these annual salaries to U.S. dollars using published exchange rates [46]. We used the weekly work hours listed in each countries’ United Nations General Service category salary scale to determine the wages per minute by using a 52-week year. For example, Syria’s salaries are based upon a 36-h work week [44], so we divided annual wages by 112,320 min (52 weeks x 36 h per week x 60 min) to obtain the salary per minute.

The estimated DOT worker labor-time for providing each dose of 3HP was obtained from CDC data describing public health worker time (9.5 min) for administering and documenting each dose of 3HP [47]. For domestic labor costs, we assumed that DOT would be provided by licensed practical nurses [39, 47], and used national average wages from the Bureau of Labor Statistics [48]. The hourly wage was divided by 60 in order to estimate the cost per minute for each worker administering 3HP DOT to patients.

### Refugee opportunity costs

Refugee time was not considered in overseas costs because refugees in camps are disallowed from salaried work, sometimes for years, while waiting to depart for the United States or another country. Undoubtedly, refugee presence in camps in any country represented some sort of consumption (food, medical care) and economic activity to the country where refugees waited. However, this economic activity was not comparable to that of residents or citizens. Refugees generally did not work, and many had few resources, so their consumption consisted of food, clothing, and shelter provided by a larger organization.

We also did not calculate refugee opportunity costs after the refugees arrived in the United States. Accordingly, in a manner similar to a previous economic evaluation, opportunity costs were not included for the patients being treated for LTBI [33].

### Cost of medications

At the time of this study, isoniazid was widely available internationally, so we used global drug facility prices to determine the cost of isoniazid for both overseas and domestic LTBI treatment. Rifapentine was not widely available internationally; for example, it was not listed in either the Global Drug Facility catalogue [49] or the Management Sciences for Health International Drug Price Indicator Guide [50]. Therefore, we determined rifapentine costs using the most recent prices made available to the federal government by the manufacturer ($1.00 per pill and $72.00 for the entire regimen) [51].

### Costs of treating active TB

All individuals who developed active TB in the United States were assumed to be treated domestically with the cost accruing to the United States health care system. The costs of treating active TB was derived from recent a recent study and updated to 2012 dollars [52, 53].

### Sensitivity analysis

We conducted one-way sensitivity analyses on several model parameters. In our first sensitivity analysis, we doubled the price for rifapentine to $2 per pill. This is similar to the price seen in a previous study that evaluated the cost of rifapentine in LTBI treatment regimens [39, 47]. We also varied the overseas costs of the TST screening from $3.50 to $5.15. This corresponded with the highest and lowest prices found after applying the country specific national price levels [42] to data for the price of a TST administered by panel physicians in Kenya ($4.80). We also varied TST specificity from 60 to 98 % in sensitivity analysis. This corresponded with the potential range of TST specificity based upon the administration and timing of the BCG vaccine [34]. We also included sensitivity analysis where we assumed that 77 % of refugees would accept 3HP overseas [38], and 82 % of refugees would complete 3HP overseas [11], as this is similar to what we used in our domestic estimates. In some cases, as many as 91 % of refugees have accepted LTBI treatment domestically [23], and as many as 91 % have also completed some type of LTBI treatment [17], so we also included these parameters in sensitivity analysis. In addition, we also included a sensitivity analysis where we did not discount the costs or benefits of outcomes occurring in the future as recommended by guidelines for conducting cost analysis in the U.S. [54].

We also included a sensitivity analysis where we added the value of a statistical life (VSL) to account for additional cost of premature mortality due to TB-related deaths. The VSL does not measure the value of an individual life, but rather the benefit due to reduced risk of mortality in a group of people. Although there is still considerable debate about the approaches used to value excess risk of mortality in economic evaluations, we believe that the VSL is an acceptable approach to use in cost-benefit analysis [55]. For modeling purposes, we estimated that 5.8 % of individuals that develop active TB die from the disease as cited in in a previous cost-effectiveness article [47]. We conservatively estimated VSL at $5.2 million based upon a lower bound estimate from the Department of Transportation [56] so that we would not overstate the benefits of the screening program due to the reduction risk of mortality.

We also included a scenario analysis where screening would take place overseas, and individuals who were TST positive overseas would be offered 3HP treatment during domestic follow-up. Individuals who were TST negative overseas would be tested again at domestic follow-up, and those individuals who were TST positive at follow-up would also be treated in the United States.

Finally, we included a scenario analysis where all refugees in the United States receiving LTBI therapy would be treated with 9 months of once-daily isoniazid. The completion rate for 9 months of isoniazid was 72 %. We averaged the completion rates for isoniazid from four studies that assessed the completion rate of isoniazid in refugees that had recently come into the U.S. [20–23]. Completed therapy with 9 months of isoniazid was assumed to have the same efficacy (93 %) as 3HP in preventing progression to active TB [11, 39].

## Results

In our analysis there were three hypothetical cohorts who tested TST positive at the following rates: high (55 %), moderate (35 %), and low (20 %). These cohorts had estimated LTBI prevalence values of 54, 27, and 7 % respectively (Table 1). We rounded the estimates of LTBI prevalence to the nearest whole number.

**Table 1:** Model parameters and assumptions for analysis comparing two programs for treating LTBI in U.S.-bound refugees

Parameter	Value	Source
Epidemiological Parameters		
Age at screening	30	Assumption
Prevalence of active TB in refugee camps (Per 100,000)		
High Prevalence	955	[28]
Moderate Prevalence	426	[28]
Low Prevalence	9	[28]
TST sensitivity	89 %	[32]
TST specificity		
Non BCG vaccinated populations	98 %	[34]
BCG vaccinated during infancy only	92 %	[34]
BCG vaccinated after infancy	60 %	[34]
Overall TST specificity^a^	85 %	calculated
Proportion with positive TST		
High Prevalence	55 %	[24–27]
Moderate Prevalence	35 %	[24–27]
Low Prevalence	20 %	[24–27]
True prevalence of LTBI^b^		
High Prevalence	54 %	calculated
Moderate Prevalence	27 %	calculated
Low Prevalence	7 %	calculated
Probability of abnormal chest radiograph with active disease	100 %	[33]
Probability of abnormal chest radiograph with LTBI (inactive TB)	11 %	[33]
Specificity of chest radiograph with no infection	95 %	[35]
Probability of accepting 12-dose weekly isoniazid-rifapentine regimen overseas with positive TST	95 %	[14]
Probability of completing 12-dose weekly isoniazid-rifapentine regimen overseas	95 %	[11, 14]
Probability of active TB during first year of resettlement for Class B1 refugees^c^	1.5 %	[36]
Probability of presenting for domestic follow-up at U.S. health department	76 %	[7]
Probability of receiving TST at domestic follow-up (Class B1)	75 %	[assumption]
Probability of receiving TST at domestic follow-up (No TB class)	50 %	[assumption]
Probability of accepting latent treatment in U.S. with positive TST	77 %	[38]
Probability of completing 12-dose weekly isoniazid-rifapentine regimen in U.S.	82 %	[11]
Effectiveness of completed 12-dose weekly isoniazid-rifapentine regimen in preventing active TB	93 %	[39]
Effectiveness of partially completed 12-dose weekly isoniazid-rifapentine regimen in preventing active TB	0 %	[39]
Annual risk of progression to active TB with untreated latent disease	0.1 %	[30]
Background Mortality	Varies with age	[23]
Cost Parameters^d^		
Costs of TST used in overseas screening with hypothetical protocol		
Base Case (average across originating countries)	$4.50	Kenyan panel physicians and [42]
Lowest TST Cost	$3.50	Kenyan panel physicians and [42]
Highest TST Cost	$5.15	Kenyan panel physicians and [42]
Cost of TST in U.S.	$24.00	[43]
Costs of 12 weekly 900 mg rifapentine doses through U.S. government	$72.00	[51]
Costs of 12 weekly 900 mg isoniazid doses through Global Drug Facility	$0.72	[49]
Costs of labor to administer DOT during latent treatment		
Average labor cost in refugee camp	$13.40	[44, 47]
U.S.	$38.70	[47, 48]
Costs of active TB case in U.S.	$18,100	[52, 53]

*BCG* Bacille Calmette-Guerin, *DOT* Directly Observed Therapy, *LTBI* latent tuberculosis infection, *TB* tuberculosis, *TST* tuberculin skin test, *U.S.* United States;

^a^Assumes that refugees are approximately equally distributed between three categories affecting BCG specificity; ^b^Calculated using the following formula: (% test positive + specificity – 1)/(sensitivity + specificity −1); ^c^Class B1 refugees indicates those with abnormal chest radiograph during overseas testing, but were not diagnosed with active TB overseas; ^d^All costs reported in 2012 dollars;

### Cases of active TB prevented

Our analysis measured the cases of active TB prevented over a 20-year period and we discounted costs and health outcomes at 3 %. We incorporated discounting of health outcomes in accordance with recommended practices, and we present the discounted values which show the societal preference for receiving benefits sooner so that less weight is placed upon cases prevented further in the future [22]. The number of active TB cases prevented varied according to the level of domestic screening. In scenarios where domestic LTBI screening occurs more frequently, implementation of overseas LTBI screening prevented 370, 185, and 48 active TB cases in U.S.-bound refugees from high, moderate, and low LTBI prevalence cohorts respectively (Table 2). Where domestic screening occurred at a more moderate rate, implementation of overseas LTBI screening prevented 440, 220, and 57 cases of active disease in refugees from high, moderate, and low LTBI prevalence cohorts respectively. When domestic LTBI screening was infrequent, overseas LTBI screening programs prevented 509, 255, and 66 active TB cases in U.S. bound refugees from high, moderate, and low LTBI prevalence cohorts. We did not include secondary transmission.

**Table 2:** Net Benefit or (Cost) and Number of Tuberculosis Cases Diagnosed in the U.S. among a Cohort of 100,000 U.S. Bound Refugees over 20 Years^a,b^

Frequency of domestic LTBI screening^c^	Prevalence of LTBI in refugee population^d^	Cases with no overseas screening (Current)	Cases with overseas screening (Proposed)	Cases prevented with overseas screening	Total cost with no overseas screening	Total cost with overseas screening	Net benefit (Cost) of overseas screening
Frequent	High	632	262	370	$15,190,000	$10,568,000	$4,622,000
	Moderate	316	131	185	$8,608,000	$6,959,000	$1,649,000
	Low	82	34	48	$3,734,000	$4,286,000	($552,000)
Moderate	High	710	270	440	$15,389,000	$10,368,000	$5,021,000
(Base Case)	Moderate	355	135	220	$8,376,000	$6,577,000	$1,799,000
	Low	92	35	57	$3,181,000	$3,769,000	($588,000)
Infrequent	High	788	279	509	$15,588,000	$10,169,000	$5,419,000
	Moderate	394	139	255	$8,143,000	$6,195,000	$1,948,000
	Low	102	36	66	$2,628,000	$3,521,000	($893,000)

*LTBI* latent tuberculosis infection, *TST* tuberculin skin test, *U.S.* United States

^a^All costs in 2012 U.S. dollars; ^b^Costs and benefits discounted at 3 % annually; ^c^Class B1 refugees are those with abnormal chest radiographs in overseas screening, frequent screening indicates LTBI testing offered to all Class B1 refugees at follow up medical examinations in U.S. and 75 % of all other refugees receive LTBI testing, with moderate screening 75 % of Class B1 refugees and 50 % of all others receive LTBI testing, with infrequent testing, 50 % of Class B1 refugees receive LTBI testing and 25 % of all others receive LTBI testing; ^d^High, moderate, and low prevalence correspond to 55, 35, and 20 % TST positive respectively

### Proportion of refugees with LTBI completing treatment

**Fig. 1:**
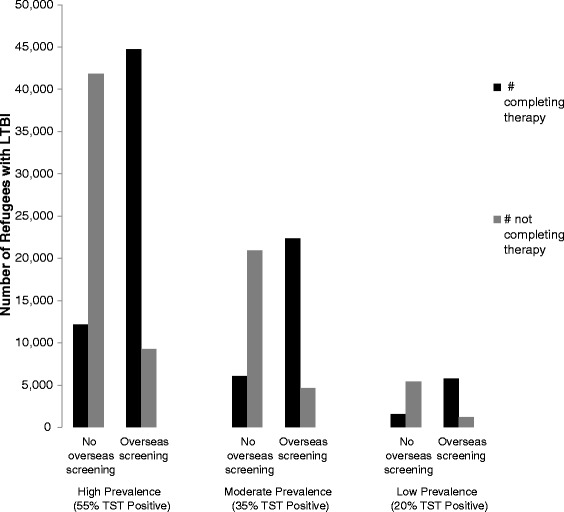
Proportion of Refugees with LTBI Completing Treatment with 12 Weekly Doses of Isoniazid and Rifapentine. LTBI = latent tuberculosis infection; TST = tuberculin skin test; With no overseas screening, all screening and treatment for LTBI takes place in the United States. With overseas screening, initial screening takes place overseas and TST positive refugees are offered treatment overseas

Overseas screening and treatment programs would treat a much higher proportion of refugees with LTBI when compared to only domestic screening and treatment (Fig. 1). For example, with overseas screening, approximately 9000 refugees with LTBI from the cohort with high LTBI prevalence would not complete 3HP therapy during the overseas screening process and domestic follow-up. However, under the current protocol, where refugees are only screened after arrival, over 40,000 would not complete 3HP treatment due to the fact that only some refugees would present for follow-up, and of these only a limited number would be able to complete treatment.

### Net costs or benefits from overseas screening

**Fig. 2:**
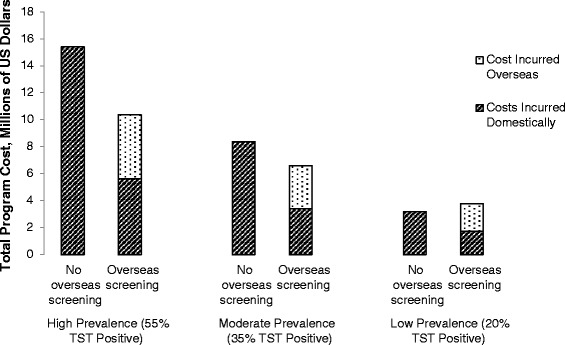
Total Cost Incurred with Two Programs for Identifying and Treating LTBI in 100,000 U.S. -Bound Refugees. LTBI = latent tuberculosis infection; TST = tuberculin skin test; U.S.  = United States. With no overseas screening, all screening and treatment for LTBI takes place in the U.S. With overseas screening, initial screening takes place overseas and TST positive refugees are offered treatment overseas. Costs incurred overseas include the TST, 12 weeks of once-weekly rifapentine and isoniazid, and labor to administer the medications. Costs incurred domestically include the TST, 12 weeks of once-weekly rifapentine and isoniazid, labor to administer the medications, and treatment of active TB patients

Overseas LTBI screening was associated with reduced costs to treat active TB in the United States, and the cost savings increased with prevalence of LTBI: $1 million (low prevalence), $4 million (moderate), and $8 million (high) (Table 3). As stated previously, total program costs included treatment of active TB cases, and resources used to screen for and treat LTBI including all supplies for TST, DOT labor, and medications. When looking at the base case with moderate levels of domestic LTBI screening, there was a net program benefit for overseas screening and treatment of refugees from high ($5.0 million benefit) and moderate ($1.8 million) LTBI prevalence populations over the 20-year evaluation period (Fig. 2). In the low LTBI prevalence population, there was a net cost of $588,000. In the case that domestic health departments screen more frequently, there is a net program benefit for overseas screening and treatment in refugees from populations with high ($4.6 million benefit) and moderate ($1.6 million benefit) LTBI prevalence and a cost of $552,000 in refugees with a low LTBI prevalence (Table 1). When domestic health departments have less frequent LTBI screening, overseas refugee LTBI screening and treatment programs resulted in a net benefits of $5.4 million and $1.9 million for high and moderate LTBI prevalence refugees respectively, while there was a cost of $893,000 for refugees with a low prevalence of LTBI.

**Table 3:** Types of Cost Incurred with Two Programs for Identifying and Treating LTBI in 100,000 U.S.-Bound Refugees^a^

	LTBI Prevalence					
	High (55 %)^b^		Moderate (35 %)^b^		Low (20 %)^b^	
	No overseas screening^c^	Overseas screening^d^	No overseas screening^c^	Overseas screening^d^	No overseas screening^c^	Overseas screening^d^
Type of cost						
Overseas LTBI						
TST	NA	$450,000	NA	$450,000	NA	$450,000
DOT Labor	NA	$669,000	NA	$426,000	NA	$246,000
Medications	NA	$3,629,000	NA	$2,312,000	NA	$1,336,000
U.S. LTBI						
TST	$941,000	$415,000	$938,000	$605,000	$937,000	$746,000
DOT Labor	$555,000	$110,000	$351,000	$118,000	$201,000	$124,000
Medications	$1,043,000	$207,000	$660,000	$222,000	$377,000	$233,000
Active TB treatment	$12,851,000	$4,887,000	$6,426,000	$2,445,000	$1,666,000	$634,000
Difference in active TB treatment^e^	$7,964,000		$3,981,000		$1,032,000	
Total program	$15,389,000	$10,368,000	$8,376,000	$6,577,000	$3,181,000	$3,769,000
Net benefit or (cost)^f^	$5,021,000		$1,799,000		($588,000)	

*DOT* directly observed therapy, *LTBI* latent tuberculosis infection, *TB* tuberculosis, *TST* tuberculin skin test, *U.S.* United States

^a^All costs in 2012 U.S. dollars; ^b^Proportion TST positive; ^c^All LTBI screening and treatment takes place in U.S.; ^d^Refugees screened with TST overseas, and TST positive refugees are offered treatment with 12 doses of once weekly rifapentine and isoniazid; ^e^Represents the reduction in U.S. incurred active TB costs with overseas screening and treatment programs; ^f^A net benefit indicates that implementing overseas screening and treatment results in cost-savings while figures enclosed in parentheses indicate that there is an additional cost associated with overseas screening and treatment

### Sensitivity analysis

In sensitivity analysis, the most influential parameters were rifapentine price, TST specificity, and the inclusion of VSL. After doubling the price of rifapentine to $2.00 a pill which was similar to that in other studies [39, 47], overseas screening had a net benefit of $2.3 million in high LTBI prevalence populations (Table 4). However, with the higher medication price, overseas screening had an additional cost of approximately $56,000 in moderate LTBI prevalence populations and $1.8 million in low LTBI prevalence populations. If the TST specificity were increased to 98 % from the baseline value of 85 %, the program would produce cost savings of approximately $5.4 million and $2.4 million in high and moderate LTBI populations respectively. With the higher specificity, the program would be nearly cost-neutral in low LTBI prevalence populations. We also included a sensitivity analysis with a VSL value of $5.2 million in order to account for the risk of premature mortality imposed by TB-related deaths. With this sensitivity analysis, overseas screening programs were cost-saving for all refugee populations: $132.7 million (high prevalence), $65.6 million (moderate prevalence), and $16.0 million (low prevalence).

**Table 4:** Sensitivity Analysis of the Net Benefit or Cost and Cases of TB Prevented from Implementing Overseas LTBI Screening in 100,000 U.S.- Bound Refugees^a,b^

	LTBI Prevalence					
	High (55 %)^c^		Moderate (35 %)^c^		Low (20 %)^c^	
	Net benefit (Cost)	Cases prevented	Net benefit (Cost)	Cases prevented	Net benefit (Cost)	Cases prevented
Parameter						
Rifapentine price doubled	$2,254,000	440	($56,000)	220	($1,767,000)	57
Overseas TST $3.50	$5,121,000	440	$1,899,000	220	($488,000)	57
Overseas TST $5.15	$4,956,000	440	$1,734,000	220	($703,000)	57
98 % TST specificity	$5,397,000	440	$2,405,000	220	$189,000	57
60 % TST specificity	$4,419,000	440	$829,000	440	($1,830,000)	57
77 % acceptance rate 3HP overseas	$3,758,000	323	$1,286,000	161	($546,000)	42
82 % completion of 3HP overseas	$4,025,000	360	$1,383,000	180	($597,000)	47
91 % acceptance rate in U.S.	$4,772,000	413	$1,680,000	207	($610,000)	54
91 % completion of 3HP in U.S.	$4,843,000	423	$1,713,000	212	($606,000)	55
No discounting of cost or benefits	$7,732,000	590	$3,155,000	295	($237,000)	76
VSL Included	$132,707,000	440	$65,641,000	220	$15,964,000	57
Screen overseas and treat in U.S.	$1,463,000	165	$288,000	83	($584,000)	21
Treat with isoniazid in U.S.	$4,493,000	458	$1,513,000	229	($694,000)	59

*LTBI* latent tuberculosis infection, *TST* tuberculin skin test; *U.S.* United States, *VSL* value of a statistical life

^a^Values reported in 2012 U.S. dollars

^b^A net benefit indicates that implementing overseas screening and treatment results in cost-savings while figures enclosed in parentheses indicate that there is an additional cost associated with overseas screening and treatment

^c^Proportion TST positive

We included a scenario analysis where we modeled patients being treated with isoniazid in the U.S. instead of 3HP. With this analysis there were net savings of $4.5 million in high LTBI prevalence populations and $1.5 million in moderate LTBI prevalence populations. There was an additional cost of $694,000 in low LTBI prevalence populations with this scenario. We also evaluated a scenario where refugees would receive initial screening overseas, but all treatment would take place in the United States. In this scenario analysis, overseas screening saved approximately $1.5 million for refugees from high LTBI populations and approximately $288,000 in refugees from moderate LTBI prevalence populations. The program resulted in additional costs of $584,000 in in low LTBI populations.

## Discussion

Implementing an LTBI screening and 3HP treatment program for refugees prior to their resettlement in the United States would decrease the numbers of infectious TB cases and would be cost-saving when implemented for refugees that had a high or moderate prevalence of LTBI. Our results show that this strategy could help make progress towards elimination of active TB in the United States, especially among refugees and foreign-born residents where domestic TB prevalence is the highest.

Currently, foreign-born individuals in the United States originating from countries with a high TB prevalence have annual TB rates exceeding 40 cases per 100,000 for many years after arrival [57, 58]. If we were not to discount the benefits of our model, after the first year we estimated an average annual TB rate of approximately 12 cases per 100,000 with overseas screening and about 42 cases per 100,000 without overseas screening in refugee populations with a high LTBI prevalence (see Additional file 1).

This study estimated the public health impact on TB reduction over a 20-year period after refugee resettlement. For example, implementation of the overseas screening in a cohort of 25,000 refugees arriving in 1 year from high LTBI prevalence regions would result in about 110 infectious TB cases prevented over a 20-year period. Similarly, in a cohort of 25,000 refugees from moderate LTBI prevalence regions, implementation would prevent about 55 infectious TB cases. Therefore, if the proposed overseas screening program were adhered to for 20 years, screening these two populations annually would prevent over 3000 active TB cases in the United States. Moreover, based on the average number of contacts as reported in a set of guidelines [59], at least 30,000 fewer people would be exposed to an individual with active, infectious TB. To be conservative, we did not consider the effect of secondary transmission on TB costs in the United States.

The total number of refugees resettling in the United States fluctuates annually, but these results indicate that overseas LTBI screening and treatment programs have the potential to prevent thousands of TB cases and would be cost-saving if used among refugees from high and moderate LTBI prevalence regions. When refugees emigrated from regions with high or moderate LTBI prevalence, we estimated that the benefits of overseas LTBI screening and treatment due to reducing the number of infectious TB cases were much greater than the costs of implementing the program. Where the program was implemented in regions with a low rate of LTBI for refugees, there was also a reduction in the active TB burden once refugees arrived in the United States, but the expenditures for program implementation were higher than the reduction in costs to treat domestic active TB cases.

This study has some limitations. First, the price of rifapentine in countries where refugees originate from was difficult to determine because the medication had not been used on a widespread basis in these countries and is not yet approved in all countries. To address this issue, we conducted a sensitivity analysis with a higher price that was similar to that seen in other studies [39, 47]. Second, the prevalence of LTBI was estimated through a fairly complex set of estimations and calculations. However, this is an approved approach that has been used in other cost-effectiveness studies [31, 32]. Because no test is available that is 100 % accurate in determining whether a person has LTBI [60], we used estimates of sensitivity and specificity obtained from the literature. Third, comparing costs and pricing (e.g., prices for goods or wages for labor) across countries is always problematic because of currency differences, differential inflationary/deflationary trends, and cultural perceptions of value. However, we used an across country equivalency value of PPP recognized by WHO and other international organizations. It was not possible to calculate the opportunity costs of refugees overseas since many countries prohibit refugees from working in the formal economy. We omitted opportunity costs for refugees both overseas and after arrival in the United States. This should lead to conservative estimates of the economic efficiency of overseas interventions because the opportunity cost of refugee time is almost certainly greater after they arrive in the United States than in the countries from which they travel. We also did not have reliable information on the proportion of refugees that had received BCG vaccination. This has an important bearing on our analysis, as an increase in the proportion of refugees with BCG vaccine would lead to more false positives due to decreased TST specificity [34]. In order to explore the potential impact of varying prevalence levels of BCG vaccine, we included a sensitivity analysis where we modeled relatively low and high levels of TST specificity.

In this study, we only considered screening with TST although the interferon-gamma release assays (IGRAs) are available. IGRAs have a higher specificity [34, 61, 62], and their use results in fewer false-positives among persons immunized with Bacillus Calmette-Guérin (BCG) vaccine, one of the most frequently administered vaccines worldwide. In general, IGRAs are more expensive to perform than TSTs [63]. To assess the impact of IGRA’s costs on the proposed screening program, we estimated a price at which the benefit of reduced false-positives would outweigh the additional costs of IGRA. In order for overseas screening to remain cost-saving, the price of the IGRA overseas would have to be less than $55 in the high LTBI prevalence populations and less than $23 in the moderate prevalence populations. A more comprehensive analysis of the costs of IGRA overseas and the impact on the cost-effectiveness of LTBI screening for these refugee populations is beyond the scope of the current paper; however, future research needs to address this issue.

In this analysis we evaluated the 12-dose weekly isoniazid-rifapentine regimen although other regimens like four months of once daily rifampin might also conceivably be considered [11]. We focused on the 12-dose weekly isoniazid-rifapentine regimen for three reasons: 1) It is most likely to be completed in the short time span between the medical screenings and departure; 2) The 12-dose weekly isoniazid-rifapentine regimen has the highest completion rates so it is most likely to have the greatest public health impact in terms of future TB cases prevented [11]; and 3) The 12-dose weekly regimen would be logistically easier to administrate overseas in refugee camps than once daily rifampin.

We found that LTBI screening and treatment among refugees from moderate and high prevalence regions would have considerable public health and economic benefits. For refugees, such a program would reduce their risk of developing active TB in the United States. With the disease’s associated out-of-pocket and opportunity costs, this would result in one less obstacle to creating a new life in a country known for offering foreign persons a new beginning. For the United States, the proposed program would save costs relative to the existing program and support CDC’s goal of TB elimination.

## Conclusions

This study assessed the additional costs and benefits associated with adding screening for LTBI to the screening that is already conducted for the screening done for active, infectious TB. Screening for and treating LTBI was logistically difficult in the past due to the limited time frame between the refugees medical screening and departure, however it is now more feasible with the 12-dose weekly isoniazid-rifapentine regimen. Most of the benefits of the program in terms of averted cases of TB occurring in the U.S. would be realized in the years succeeding the refugee’s resettlement in the U.S. As such, implementation of the program would represent an initial investment that would yield cost savings over time, particularly for refugees from areas with a relatively high or moderate prevalence of LTBI. In terms of public health impact, the program would also be most efficient if it focused on refugee from areas with a high prevalence of LTBI under a variety of scenarios.

## Additional file

Additional file 1:
**Technical appendix refugee LTBI screening.** (DOCX 437 kb)
