# Supplementary material for: A cost-benefit analysis of a proposed overseas refugee latent tuberculosis infection screening and treatment program
Source: BMC Public Health. 2015 Dec 1;15:1201. doi: 10.1186/s12889-015-2530-7 (PMC4666176; doi:10.1186/s12889-015-2530-7)
Supplement: Additional file 1: — Technical appendix refugee LTBI screening. (DOCX 437 kb) [file 12889_2015_2530_MOESM1_ESM.docx]

**Technical Appendix: A cost-benefit analysis of a proposed overseas refugee latent tuberculosis infection screening and treatment program**

La’Marcus T. Wingate, Margaret S. Coleman, Christopher de la Motte Hurst, Marie Semple, Weigong Zhou, Martin S. Cetron, John A. Painter

Corresponding Author: La’Marcus T. Wingate : [latewing@gmail.com](mailto:latewing@gmail.com)

**Disclaimer:** The findings and conclusions in this manuscript are those of the authors and do not necessarily represent the official position of the Centers for Disease Control and Prevention or the U.S. Department of Health and Human Services.

**Section 1: Overview of decision tree and Markov modelling**

Figure 1 depicts an overview of the decision tree used to compare the costs and benefits of two protocols for treating latent tuberculosis infection (LTBI) in U.S.-bound refugees. All refugees are in one of three conditions at the beginning: active tuberculosis (TB) disease, LTBI, or no tuberculosis infection. The current protocol incorporates screening for active TB disease with a chest radiograph overseas. The vast majority of refugees with active TB disease at the time of screening are assumed to be diagnosed overseas and treated to cure before departure. However, refugees with LTBI or no TB infection may also have an abnormal chest radiograph overseas, yet not be diagnosed with active TB. In this case the refugee receives a Class B1 TB designation.^1^ It is important to note that no LTBI screening takes place overseas under the current protocol. The proposed protocol is similar to the current protocol with the notable exception that LTBI screening takes place overseas via administration of a tuberculin skin test (TST) for all refugees at the same time as the chest radiograph.


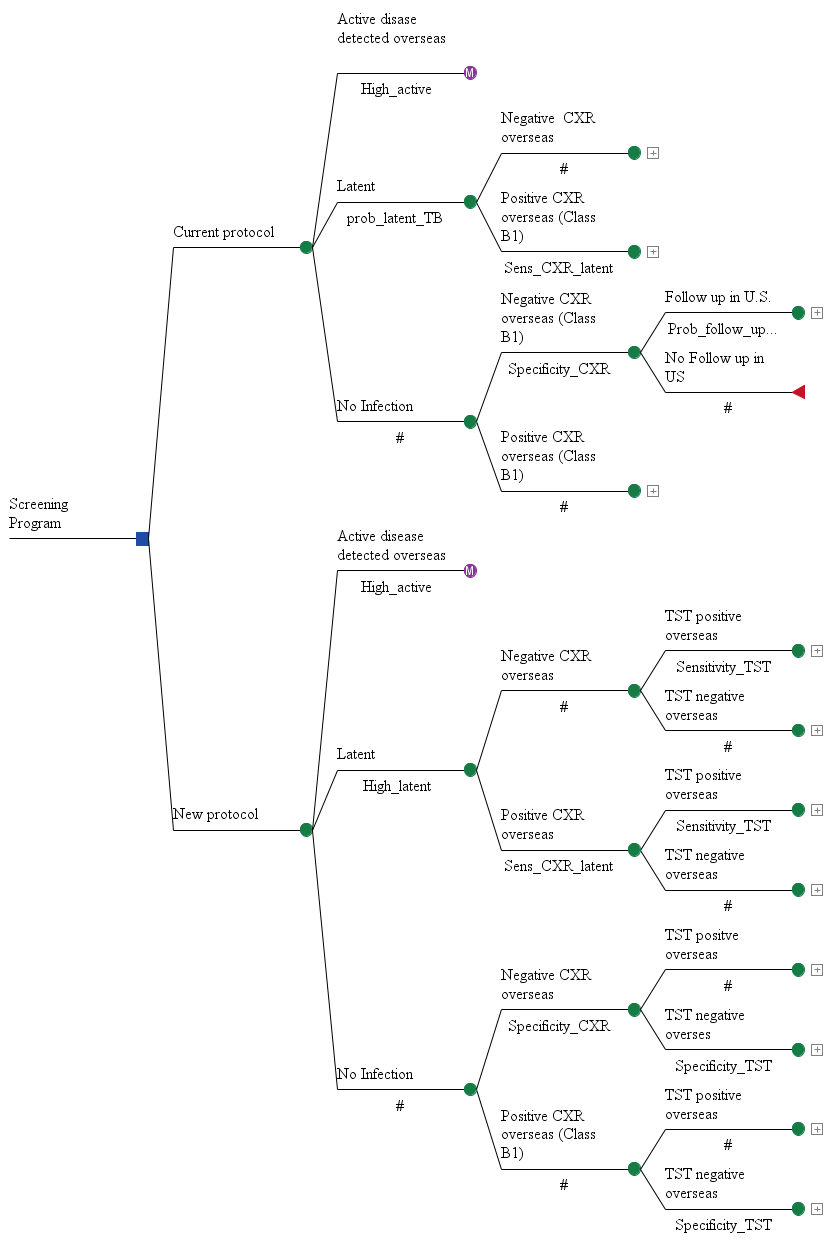


Figure 1: Overview of decision tree for comparison of two protocols for screening treating LTBI in U.S. bound refugees

All refugees are encouraged to present to their receiving state or local health departments for follow-up medical evaluations after U.S. arrival. Figure 2 presents a diagram of how screening for LTBI takes place in the current protocol during this follow-up. As shown in this portion of the decision tree, there is a probability that some refugees would not present for these medical evaluations.^2^ For those refugees that do present for follow-up examinations in the U.S., there is a chance that some of them may not be screened for LTBI. If refugees are screened for LTBI, there is a chance they could have a positive or negative test. If the TST is positive, then refugees may either decline or accept treatment. In our base case analysis, the refugee is offered treatment with a 12 week isoniazid rifapentine regimen (3HP). If the refugee accepts treatment, there is a chance that some refugees may not complete treatment.


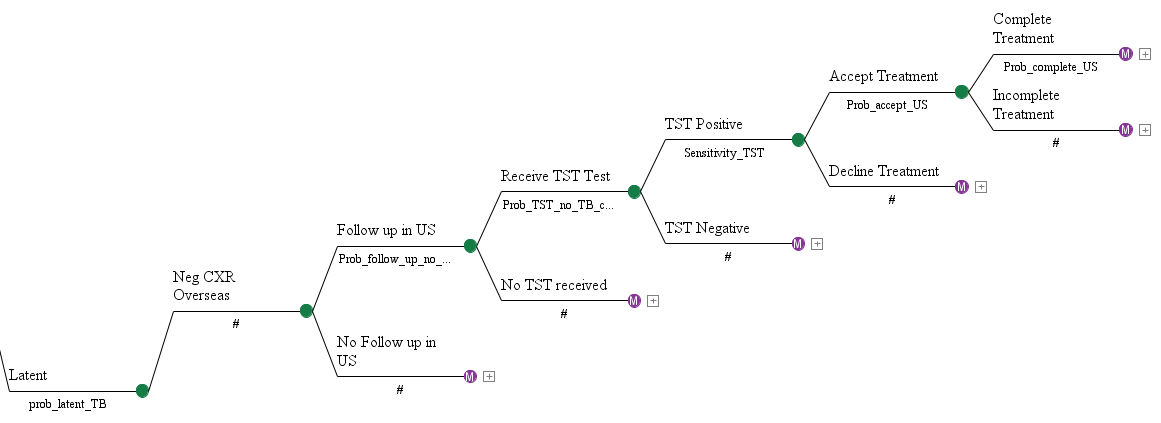


Figure 2: LTBI screening for U.S. bound refugees under the current protocol of no overseas screening or treatment for latent tuberculosis infection

Figure 3 presents a diagram of how LTBI screening takes place under the proposed protocol. As with the current protocol, all refugees receive a chest radiograph overseas to screen for active TB, but the proposed protocol implements screening for LTBI overseas as well using the TST test. If refugees have a positive TST overseas, they are offered treatment with 3HP at that time. Refugees have the opportunity to either decline or accept treatment overseas. Previous data indicates that the acceptance and completion rates for refugees overseas with presumptive treatment for intestinal parasites, a voluntary program, is very high.^3^ In our base case analysis we assume that five percent of refugees are unable to complete therapy overseas due to side effects.^4^ Accordingly, with overseas screening and treatment, there will be three primary reasons why individuals do not complete treatment overseas: pregnancy, medical contraindications to staring therapy, or a side effect that would preclude them from completing therapy. Therefore, there would be no need for rescreening of LTBI in the U.S. A refugee could also have a negative TST test overseas. In the event that a refugee has a negative LTBLI test overseas, they would be encouraged to proceed to follow-up medical evaluations in the U.S. with a chance for subsequent testing and treatment.


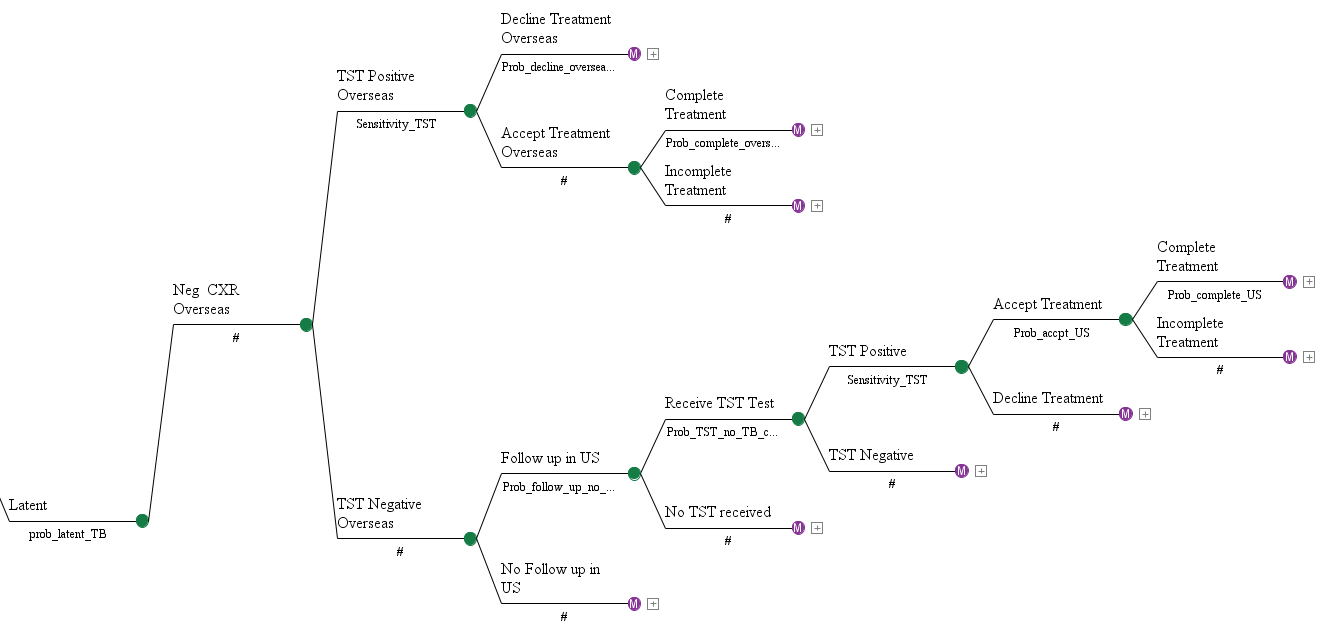


Figure 3: LTBI screening and treatment for U.S. bound refugees under the proposed protocol incorporating overseas screening and treatment for latent tuberculosis infection

Figure 4 depicts the Markov node for an individual with untreated LTBI and an abnormal chest radiograph overseas (Class B1). This applies to the current and proposed protocols. For Class B1 refugees, a small portion (1.5%) have imported TB during their first year in the U.S, and everyone else is in the untreated LTBI state at the beginning.^5^ Many of the imported TB cases within the first year are likely cases that were present at the time the refugee was overseas. Refugees with untreated LTBI disease have an annual probability of 0.1% to convert to active TB.^6,7^ For individuals that complete treatment with 3HP, their chance of converting to active TB is reduced by 93%.^8^

The Markov nodes for individuals with untreated latent disease and a normal chest radiography overseas are identical with the exception that all refugees start off in the untreated latent state. The probability of conversion to active TB is also 0.1% for these refugees and the 3HP is 93% effective.

With the current protocol a refugee with LTBI could remain untreated for several reasons after U.S. arrival: failure to present for follow up medical evaluations, not being offered a TST during follow up, a negative TST at medical follow up, declining treatment, or incomplete treatment. With the proposed protocol, a refugee with LTBI could receive a positive TST overseas yet remain untreated by declining treatment or failing to complete treatment. Under the proposed protocol, a refugee with LTBI and a negative TST overseas could remain untreated by failing to present for follow-up evaluations, not receiving a TST at follow-up, a negative TST during medical follow-up, declining treatment, or incomplete treatment. The reasons for remaining untreated apply to Class B1 refugees and those with no TB class.


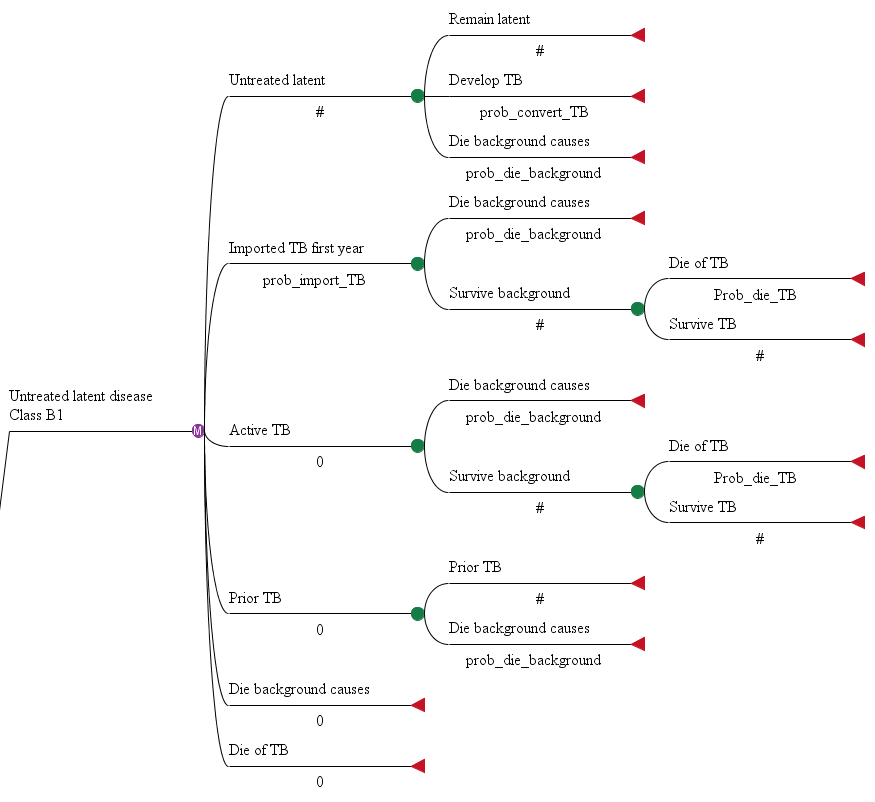


Figure 4: Markov node for individuals with untreated LTBI

**Section 2: Data sources for major epidemiological parameters**

*Prevalence of active TB in refugee camps*

The prevalence of active TB diagnosed in refugee camps was sourced from the Migration Health Report produced by the International Organization of Migration.^9^ The handbook contains data from the results of medical examinations conducted in refugee camps overseas. Refugees in regions with the highest prevalence of active TB had 955 cases per 100,000, and refugees hailing from regions that had the second highest prevalence of disease had 426 cases per 100,000. Meanwhile, refugees from areas with the lowest prevalence of active TB had 9 cases per 100,000.

*Derivation of TST Specificity*

TST specificity varies as a function of BCG vaccination, wherein higher levels of BCG vaccination and later timing of BCG in a population are associated with lower levels of TST specificity. More specifically, when the BCG vaccine is given in infancy, the TST specificity is approximately 92%. When the BCG vaccine is given after the age of one, TST specificity decreases to 60%. When there is no BCG vaccine given, the TST specificity is 98%.^10^ We had no data available on the proportion of refugees that had received BCG vaccine, nor any data on when they had received the vaccine, so we assumed that refugees were divided approximately equally between no BCG vaccination, BCG vaccination received during infancy, and BCG vaccination received after infancy. The average specificity in these situations is 83.3%, but we rounded it to 85% as an estimate. Given that there was substantial uncertainty regarding this parameter, we included it in sensitivity analysis with ranges of 60% to 98% in order to reflect the minimum and maximum specificities TST resulting from BCG vaccination.

*Proportion of refugees with positive TST*

In order to determine a plausible range of refugees that might test positive with the TST, we searched the literature for studies that evaluated the proportion of refugees with positive TST results in the U.S. In their study of refugees arriving into Minnesota, Varkey and colleagues found that the percentage of refugees with a positive TST result ranged from 40% to 55% depending upon the refugee’s continent of origin.^11^ Chai and colleagues found that 38% of refugees coming into the Washington D.C. area had a positive TST result,^12^ while Kowatsch-Beyer and colleagues indicated that approximately 46% of refugees arriving in Ohio had positive TST results.^13^ A study by Bennett and colleagues utilized data from the National Health and Nutrition Examination Survey and indicated that 19% of the general foreign-born population had positive TST results.^14^ Based upon this data, we used figures of 55%, 35%, and 20% as plausible estimates for the proportion of refugees with positive TST results in regions with high, moderate, and low prevalence of LTBI infection.

For modeling purposes, we needed to estimate the proportion of refugees that truly had LTBI which is distinct from that number with positive TST results. In order to accomplish this, we used the following formula as demonstrated in Shah and colleagues:^15^ (% test positive + specificity – 1)/(sensitivity + specificity – 1). Using this formula, we estimated the proportion of refugees with LTBI from the high, moderate, and low LTBI prevalence populations by utilizing the region specific proportion of refugees that would have positive TST tests.

*Chest radiograph test characteristics*

We made two assumptions based on a previous cost-effectiveness study that examined screening for LTBI in populations emigrating to a new country: 1) that the chest radiograph would have 100% sensitivity for screening of active TB disease, and 2) assumed that 11% of the refugees with LTBI would have abnormal chest radiographs.^6^ These would be refugees with inactive TB disease and they would have a Class B1 designation due to their positive chest radiographs during overseas screening.

*Probability of accepting and completing 3HP regimen*

When refugees are offered voluntary presumptive treatment for intestinal parasites, the primary reason that adults do not accept treatment is because of pregnancy.^3^ In our model, we assume that the primary reasons adult refugees would not accept treatment overseas is because of pregnancy or medical contraindications. Accordingly, we estimate that 95% of refugees would accept treatment with 3HP.

However because of the uncertainty in these measurements, we included this parameter in sensitivity analysis. In sensitivity analysis, we lowered the acceptance rate to 77%. We used 77% because this is the rate of acceptance observed in the foreign-born while they are in the U.S.^16^ We assumed that the vast majority of refugees would go on to complete 3HP treatment overseas in a fashion similar to that seen with presumptive treatment for intestinal parasites.^3^ The only reason that refugees would not complete treatment overseas would be due to side effects that preclude treatment (five percent).

*3HP effectiveness*

Based upon the example of a previous cost-utility analysis, we estimated that 3HP would be 93% effective in preventing progression to active TB in refugees with LTBI.^8^ We conservatively estimated that refugees who had to cease 3HP treatment did so at two weeks with a corresponding effectiveness of 0%. This conservative estimate helps to avoid overestimating the cost of the overseas screening program since more refugees take the 3HP in the proposed program.

*Reactivation rate with LTBI*

We used an annual risk of 0.1% to model the probability of converting from LTBI to active TB. This data is derived from surveillance data of progression to TB in a group of refugees.^17^ This reactivation rate has been used in multiple cost-effectiveness analyses,^6,18,19^ and represents a conservative rate of reaction when compared to reactivation rates used in other cost-effectiveness analyses.^7^ Thus this conservative reactivation rate prevents us from overstating the benefits of the overseas screening and treatment program as more refugees would be treated with this regimen.

*Overseas TST price*

The price of the TST during overseas screening was derived by using unpublished data from Kenyan Panel Physicians. Panel physicians work in the refugee’s country of origin and are selected by the United States Department of State to administer the medical examinations overseas.^1^ The Kenyan panel physicians reported that the local price for a TST in American dollars was $4.80. In order to estimate the price of the TST in other refugee camps, we used country specific national price levels. The national price level is an index that is used when comparing the price of goods across countries with the U.S. dollar as the benchmark.^20^ As an example, during 2012, Kenya’s national price level was 0.44 (rounded to two decimals), so 44 cents would buy the same in Kenya as one dollar would in the U.S.

We downloaded the 2012 national price levels from World Bank data^20^ in order to obtain the national price levels for the countries where most U.S. bound refugees originated from.^9^ In addition to Kenya, we included Thailand, Malaysia, Nepal, Ethiopia, Uganda, Iraq, Jordan, and Syria. Syria did not have national price data listed for 2012. For the remaining countries, the national price levels ranged from 0.32 to 0.47. Accordingly, the price of the TST across these countries ranged from $3.50 to $5.15. When the price of the TST was averaged across all of the countries excluding Syria, the average price was found to be $4.49. We rounded this estimated price to $4.50. We used the range of prices found in the other countries in our sensitivity analysis in order to account for uncertainty in this parameter estimate.

*Overseas DOT Labor Cost*

The country specific cost for labor to administer 3HP was determined using the most recently published (2011-2013) United Nations (U.N.) pay scales available at the time the study was being conducted.^21^ Talks with CDC personnel familiar with overseas operations in refugee camps indicated that the salaries of medical officials in refugee camps that might administer DOT to refugees would be most similar to a U.N. General Service Category Step 3 Level 3 pay grade. We adjusted wages for the countries to a base year of 2012 using GDP deflator data obtained from the International Monetary Fund if the wage data was reported for 2011 or 2013.^22^ Our next step was to convert these salaries into a common currency of U.S. dollars, and we did this using exchange rates for 2012 from the Central Intelligence Agency World Factbook.^23^ We then utilized data from the pay scales to estimate the annual number of work hours for each respective country. The hourly salaries were used to estimate the salary per minute. For example, Syria’s salaries were based upon a 36-hour work week,^21^ so we divided annual wages by 112,320 minutes (52 weeks x 36 hours per week x 60 minutes) to obtain the salary per minute.

**Section 3: Absolute number of active TB cases diagnosed and prevented**

In our base case analysis presented in the manuscript, we discounted costs and outcomes at a 3% annual rate in accordance with recommendations for economic evaluations.^24^ However, from a public health standpoint, it is worthwhile to look at the absolute number of TB cases presented. We show the results from this analysis in Table 1 below. In this analysis, we exclude the cases that were diagnosed among Class B1 refugees shortly after U.S. entry as some of these cases may have been active cases that were imported from overseas.^25^ This analysis uses assumptions from our base case where 75% of the Class B1 refugees receive LTBI testing if they present to follow-up, and 50% of the Class B2 refugees received LTBI testing during follow-up exams. The number of cases prevented varied from 77 to 591 depending upon the prevalence of LTBI in the refugee’s country of origin.

**Table 1: Tuberculosis cases diagnosed in the U.S. among a cohort of 100,000 U.S.-bound refugees over 20 years**

|  | LTBI prevalence | | | | | |
| --- | --- | --- | --- | --- | --- | --- |
|  | High (55%)^A^ | | Moderate (35%)^A^ | | Low (20%)^A^ | |
|  | No overseas screening^B^ | Overseas screening^C^ | No  overseas screening^B^ | Overseas screening^C^ | No  overseas screening^B^ | Overseas screening^C^ |
| **No discounting** |  |  |  |  |  |  |
| Total cases | 834 | 243 | 417 | 122 | 109 | 32 |
| Cases prevented | 591 | | 295 | | 77 | |

LTBI=latent tuberculosis infection; TST=tuberculin skin test

A: Proportion TST positive; B: All LTBI screening and treatment takes place in the U.S.; C: Refugees screened with TST overseas, and TST positive refugees are offered treatment with 12 doses of once weekly rifapentine and isoniazid

**Section 4: Monte Carlo multivariate probabilistic sensitivity analysis**

A Monte Carlo probabilistic sensitivity analysis was performed to further evaluate the effect of uncertainty. A Monte Caro probabilistic sensitivity analysis allows for an assessment of the outcomes when multiple parameters are varied simultaneously. This type of sensitivity analysis allows for random sampling of parameter values from distributions that are created to represent plausible values for the chosen variables. The Monte Carlo probabilistic sensitivity analysis was performed with 10,000 iterations.

**Table 2: Parameter values used in Monte Carlo probabilistic sensitivity analysis**

| **Parameter** | Distribution | Distribution characteristics |
| --- | --- | --- |
| **Epidemiological parameters** |  |  |
| TST specificity | Beta | 85% mean |
| Probability of declining a 12-dose weekly isoniazid-rifapentine regimen overseas with positive TST | Beta | 5% mean |
| Probability of completing 12-dose weekly isoniazid-rifapentine regimen overseas | Beta | 95% mean |
| Probability of presenting for domestic follow-up at U.S. health department | Beta | 76% mean |
| Probability of receiving TST at domestic follow-up (Class B1) | Beta | 75% mean |
| Probability of receiving TST at domestic follow-up (No TB class) | Beta | 50% mean |
| Costs of TST used in overseas screening with hypothetical protocol | Triangular | $3.30 minimum, $5.15 maximum, most likely $4.50 |
| Costs of 12 weekly 900 mg rifapentine doses | Uniform | $72.00 minimum, $144.00 maximum |

Figure 5 shows the proportion of times that the new protocol is the optimal selection when compared to the current protocol when evaluating the Monte Carlo probabilistic sensitivity analysis for high LTBI prevalence populations. For the purposes of this particular analysis, a strategy is deemed the optimal selection whenever it has lower overall costs than the alternative program. As seen in Figure 5, the new protocol that incorporates overseas screening and treatment has lower overall costs nearly 100% of the time.


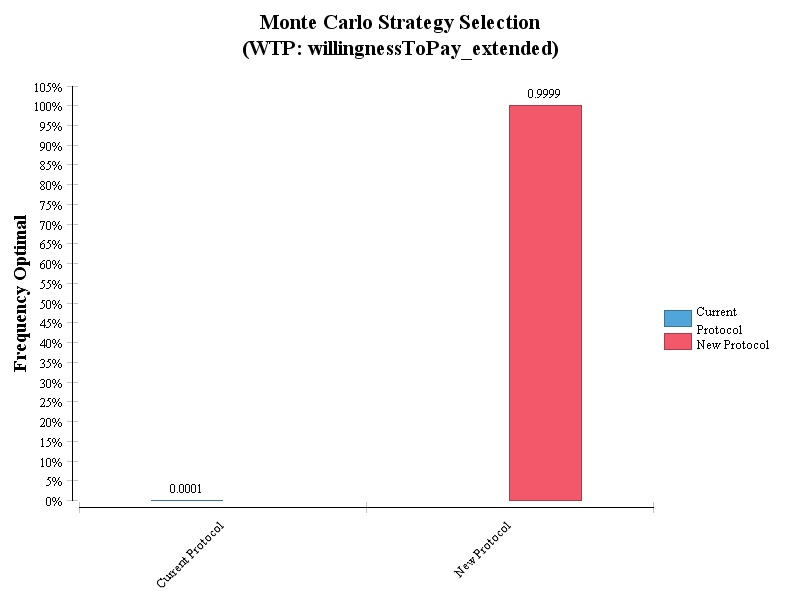


Figure 5: Proportion of times each protocol is the optimal selection for screening and treatment of LTBI in refugees from high LTBI prevalence populations

Figure 6 shows the percentage of times that the proposed protocol is the optimal selection when compared to the current protocol when using the results of the Monte Carlo probabilistic sensitivity analysis for refugee populations with a moderate prevalence of LTBI. A protocol is deemed optimal whenever it has lower costs compared to the other protocol. The new protocol was the optimal protocol in nearly 70% of the iterations obtained from the Monte Carlo probabilistic sensitivity analysis.


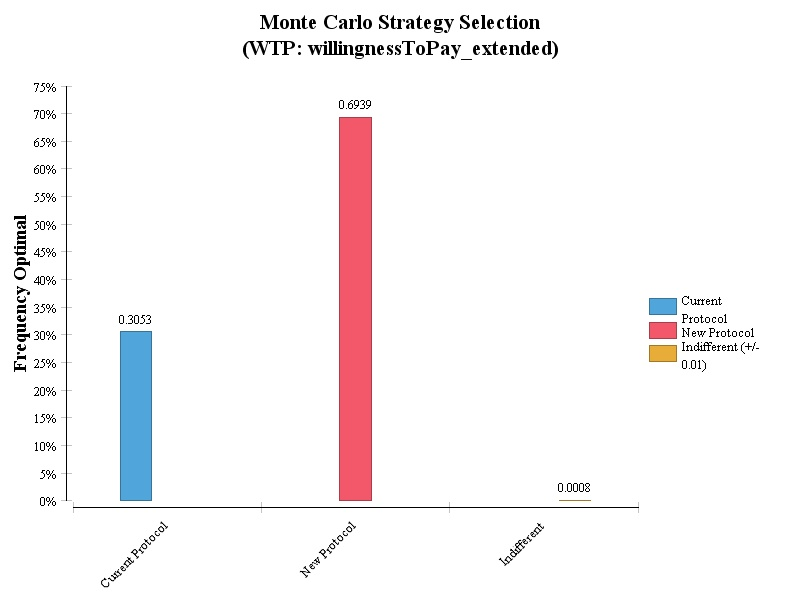


Figure 6: Proportion of times each protocol is the optimal selection for screening and treatment of LTBI in refugees from moderate LTBI prevalence populations

Figure 7 is a graphical depiction of the percentage of times that the proposed protocol is the optimal selection when compared to the current protocol for low LTBI prevalence refugee populations when using the Monte Carlo probabilistic sensitivity analysis. The protocol with lover overall costs is the optimal protocol. The current protocol was the optimal protocol in virtually all of the simulatoins for this population.


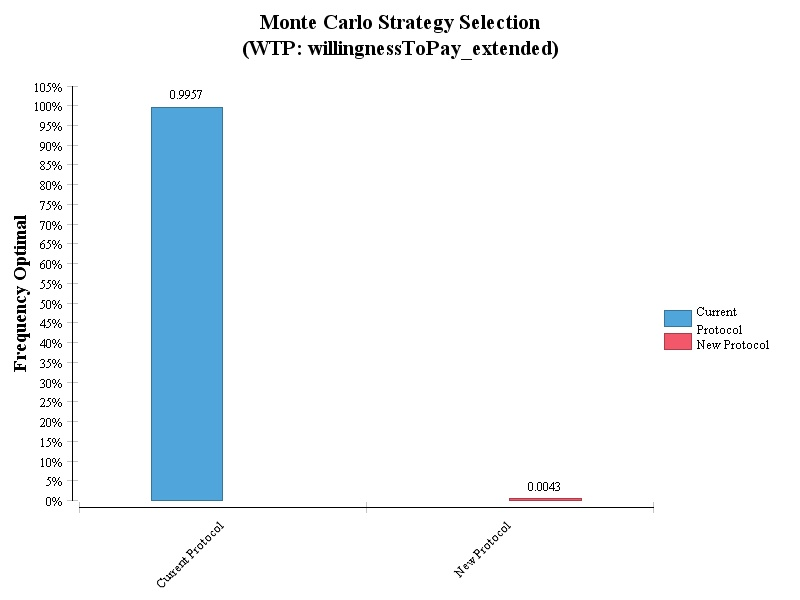


Figure 7: Proportion of times each protocol is the optimal selection for screening and treatment of LTBI in refugees from moderate LTBI prevalence populations

**Section 5: Explanation of alternative scenarios**

Figure 8 depicts the decision tree that is used in the scenario analysis where INH is used to treat refugees in the U.S. The sequence of events is similar to that seen in the base case analysis, and the primary difference is the form of treatment in the U.S. However, 3HP is used during overseas treatment.


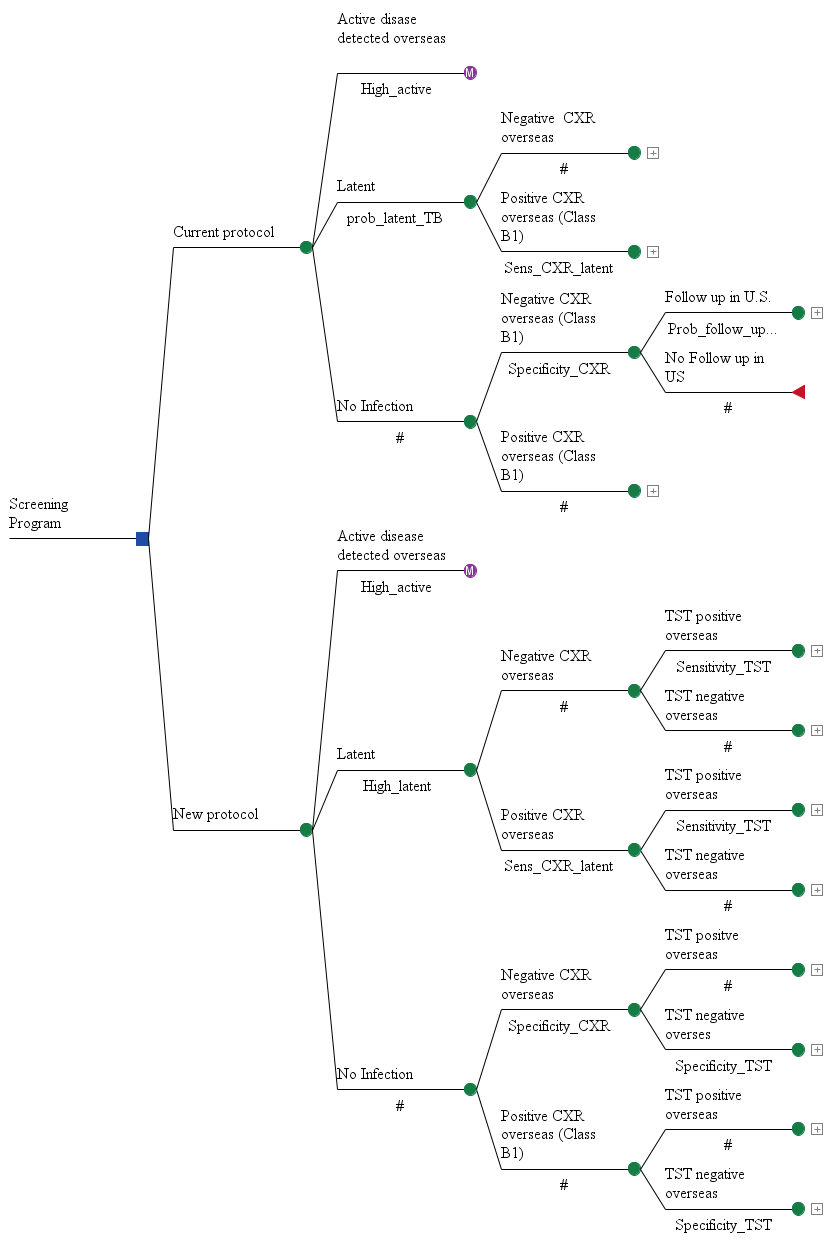


Figure 8: Overview of decision tree for comparison of two protocols for treating LTBI in U.S. bound refugees where INH is used for treatment in the U.S.

Figure 9 depicts part of the decision tree under the alternative proposed protocol where screening for LTBI is conducted overseas, but all treatment takes place in the U.S. This portion of the decision tree demonstrates the sequence of events for refugees with a positive TST overseas. All refugees are encouraged to present for follow up medical evaluations once they arrive in the U.S. Refugees that present for follow up evaluations and have a prior positive TST from overseas are offered domestic treatment with 3HP in the U.S.


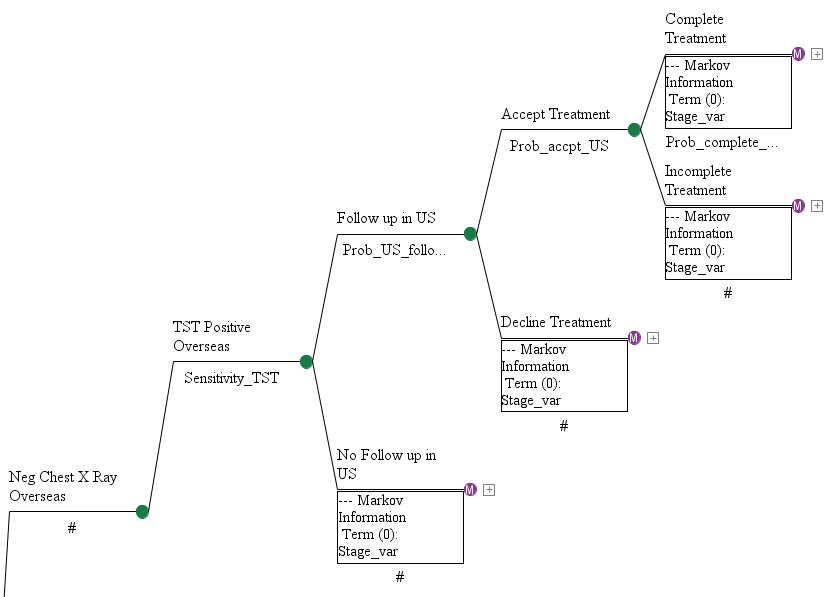


Figure 9: LTBI screening for U.S. bound refugees with a positive TST overseas under the alternative proposed protocol incorporating screening overseas and treatment in the U.S.

Figure 10 depicts the part of the decision tree under the alternative proposed protocol where screening for LTBI is conducted overseas, but all treatment takes place in the U.S. The diagram shows the sequence of events for refugees with a negative TST overseas. Refugees are encouraged to present for follow up medical evaluations after U.S. arrival. Refugees presenting for follow up may or may not receive LTBI testing again if they had a negative TST result during overseas testing.


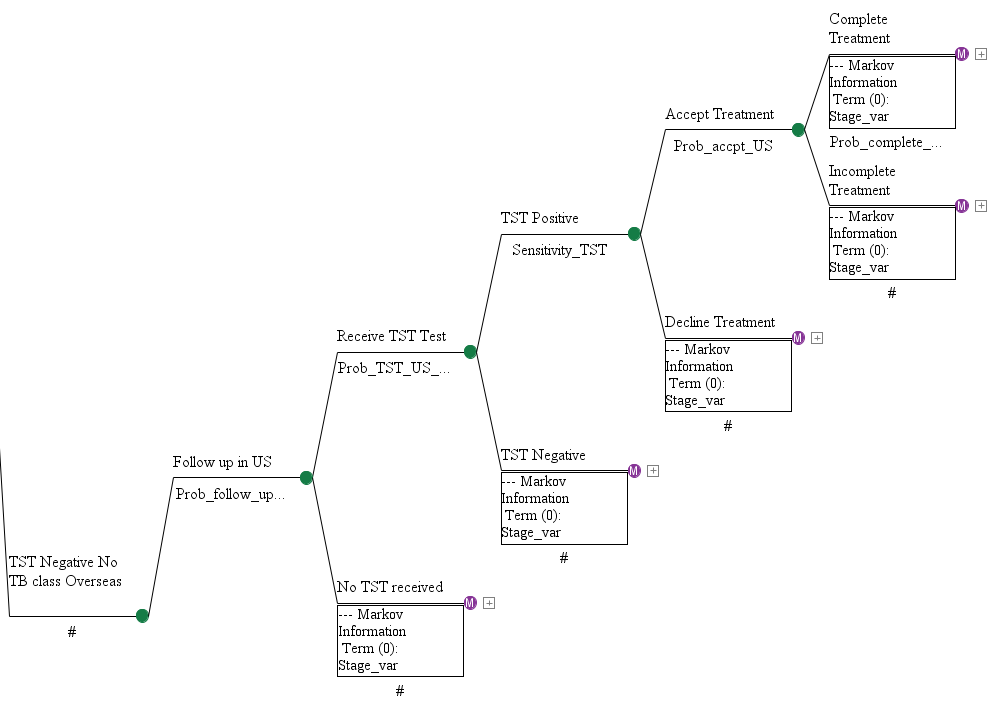


Figure 10: LTBI screening for U.S. bound refugees with a negative TST overseas under the alternative proposed protocol incorporating screening overseas and treatment in the U.S.

**References**

1. Centers for Disease Control and Prevention: Tuberculosis screening and treatment technical instructions (TB TIs) using cultures and directly observed therapy (DOT) for panel physicians 2013. <http://www.cdc.gov/immigrantrefugeehealth/exams/ti/panel/tuberculosis-panel-technical-instructions.html> (2013). Accesed 2 Nov 2013.

2. Liu Y, Weinberg MS, Ortega LS, Painter JA, Maloney SA. Overseas screening for tuberculosis in U.S.-bound immigrants and refugees. N Engl J Med*.* 2009;360(23):2406-15.

3. Shah JJ, Maloney SA, Liu Y, Flagg EW, Johnston JP, Young SA, Weston R, et al. Evaluation of the impact of overseas pre-departure treatment for infection with intestinal parasites and Montagnard refugees migrating from Cambodia to North Carolina. Am J Trop Med Hyg. 2008;78(5):754-9.

4. Sterling TR, Villarino ME, Borisov AS, Shang N, Gordin F, Bliven-Sizemore E, et al. Three months of rifapentine and isoniazid for latent tuberculosis infection. N Engl J Med*.* 2011;365(23):2155-66.

5. Lowenthal P, Westenhouse J, Moore M, Posey DL, Watt JP, et al. Reduced importation of tuberculosis after the implementation of an enhanced pre-immigration screening protocol. Int J Tuberc Lung Dis. 2011;15:761-6.

6. Oxlade O, Schwartzman K, Menzies D. Interferon-gamma release assays and TB screening in high-income countries: A cost-effectiveness analysis. Int J Tuber Lung Dis. 2007;11(1):16-26.

7. Oxlade O, Pinto M, Trajman A, Menzies D. How methodologic differences affect results of economic analyses: a systematic review of interferon gamma release assays for the diagnosis of LTBI. PloS One*.* 2013;8(3):e56044.

8. Holland DP, Sanders GD, Hamilton CD, Stout JE. Costs and cost-effectiveness of four treatment regimens for latent tuberculosis infection. Am J Respir Crit Care Med. 2009;179(11):1055-60.

9. International Organization for Migration. Migration health annual review 2012. http://publications.iom.int/bookstore/free/MHD_AR_2012_11Sept2013.pdf (2013). Accessed March 17, 2015.

10. Farhat M, Greenaway C, Pai M, Menzies D. False-positive tuberculin skin tests: What is the absolute effect of bcg and non-tuberculous mycobacteria? Int J Tuber Lung Dis. 2006;10(11):1192-204.

11. Varkey P, Jerath AU, Bagniewski SM, Lesnick TG. The epidemiology of tuberculosis among primary refugee arrivals in minnesota between 1997 and 2001. J Travel Med. 2007;14(1):1-8.

12. Chai SJ, Davies-Cole J, Cookson ST. Infectious disease burden and vaccination needs among asylees versus refugees, district of columbia. Clin Infect Dis*.* 2013;56(5):652-8.

13. Kowatsch-Beyer K, Norris-Turner A, Love R, Denkowski P, Wang SH. Utilization of a latent tuberculosis infection referral system by newly resettled refugees in central Ohio. Int J Tuberc Lung Dis. 2013;17(3):320-5.

14. Bennett DE, Courval JM, Onorato I, Agerton T, Gibson JD, Lambert L, et al. Prevalence of tuberculosis infection in the united states population: The national health and nutrition examination survey, 1999-2000. Am J Respir Crit Care Med. 2008;177(3):348-55.

15. Shah M, Miele K, Choi H, DiPietro D, Martins-Evora M, Marsiglia V, et al. Quantiferon-TB gold in-tube implementation for latent tuberculosis diagnosis in a public health clinic: A cost-effectiveness analysis. BMC Infect Dis. 2012;12:360.

16. Horsburgh CR, Jr., Goldberg S, Bethel J, Chen S, Colson PW, Hirsch-Moverman Y, et al. Latent TB infection treatment acceptance and completion in the United states and Canada. Chest*.* 2010;137(2):401-9.

17. Nolan CM, Elarth AM. Tuberculosis in a cohort of Southeast Asian Refugees. A five year surveillance study. Am Rev Respir Dis. 1988; 137(4):805-9.

18. Tan M, Menzies D, Schwartzman K. Tuberculosis screening of travelers to higher-incidence countries: a cost-effectiveness analysis. BMC Public Health. 2008;8:201.

19. Schwartzman K, Menzies D. Tuberculosis screening of immigrants to low-prevalence countries. A cost-effectiveness analysis. Am J Respir Crit Care Med. 2000;161:780-9.

20. The World Bank Group. World development indicators: exchange rates and prices. [Http://wdi.Worldbank.Org/table/4.16#](Http://wdi.Worldbank.Org/table/4.16) (2014). Accessed 27 Aug 2015.

21. Salary Scales for Staff in the General Service and Related Categories. United Nations, New York. 2013. http://www.un.org/Depts/OHRM/salaries_allowances/salaries/gs.htm. Accessed 5 Nov 2013.

22. World Economic Outlook Database. International Monetary Fund, Washington D.C. 2013. [Http://www.Imf.Org/external/pubs/ft/weo/2012/01/weodata/index.Aspx. Accessed 5 Nov 2013](Http://www.Imf.Org/external/pubs/ft/weo/2012/01/weodata/index.Aspx.%20Accessed%205%20Nov%202013).

23. The World Factbook. Central Intelligence Agency, Washington D.C. 2013. Https://[www.Cia.Gov/library/publications/the-world-factbook/](http://www.Cia.Gov/library/publications/the-world-factbook/). Accessed 14 Jan 2014.

24. Corso PS, Haddix AC. Time effects. In: Haddix AC, Teutsch SM, Corso PS, editors. Prevention effectiveness a guide to decision analysis and economic evaluation. New York: Oxford university press; 2003. p. 92-102.

25. Walter ND, Painter J, Parker M, Lowenthal P, Flood J, Fu Y, et al. Persistent latent tuberculosis reactivation risk in United States immigrants. Am J Respir Crit Care Med*.* 2014;189(1):88-95.
